# Unfulfilled Needs in the Detection, Diagnosis, Monitoring, Treatment, and Understanding of Glaucoma in Blacks Globally

**DOI:** 10.1007/s40615-023-01679-2

**Published:** 2023-06-20

**Authors:** Ayobami Adebayo, Daniel Laroche

**Affiliations:** 1 Albert Einstein College of Medicine, Bronx, NY USA; 2 New York Eye and Ear Infirmary of Mount Sinai, New York, NY USA

**Keywords:** Glaucoma, Reparations, Ophthalmology, Disparities, Cataract

## Abstract

Glaucoma is an ophthalmic disorder that affects a significant number of Blacks globally. A leading cause of this condition is an age-related enlargement of the lens and increased intraocular pressure. Although Blacks are affected by glaucoma at a higher rate than their Caucasian counterparts, there remains a lack of emphasis placed on the detection, diagnosis, monitoring, and treatment of glaucoma in this population. Education regarding glaucoma in the African and African American populations is essential to reducing rates of glaucoma-related visual impairment and improving treatment success. In this article, we highlight specific issues and limitations to the management of glaucoma, which affects Blacks at a higher rate. In addition, we also review the backgrounds of Blacks globally and examine historical events that have contributed to financial inequality and wealth/health disparities affecting glaucoma management. Lastly, we suggest reparations and solutions that health care professionals can use to improve glaucoma screening and management.

## Background

Glaucoma is a common ophthalmic disorder that affects many people and is distinguished by the loss of retinal ganglion cells, the excavation of the optic nerve, and progressive loss of sight [1]. A leading cause of this condition is an age-related enlargement of the lens and increased intraocular pressure (IOP); however, there are a variety of different contributors to the condition. Glaucoma is a prominent cause of permanent blindness across the globe. Its prevalence in the USA is approximately 1900 per 100,000 persons aged over 40 years, and the ailment is responsible for over 10 million physician visits each year [2]. The yearly cost of treating eye issues related to glaucoma in the USA is estimated at $2.5 billion [3]. Glaucoma is a significant public health concern, especially for African Americans. According to the Baltimore Eye Survey, the prevalence of glaucoma in African Americans aged 50–59 is about 4%. This number increases to 13% in African Americans aged 80–89. African Americans are three to four times more likely to have a diagnosis of glaucoma when compared to their Caucasian counterparts as they age [4]. Globally, Africa has the highest prevalence of blindness from glaucoma in comparison to other continents of the world. Glaucoma accounts for 15% of blindness in this region [5]. Additionally, open-angle glaucoma is the leading cause of blindness in the Caribbean. In Barbados, open-angle glaucoma accounts for 28.4% of cases of blindness [6]. Although there are no published prevalence data from Jamaica, the prevalence of glaucoma is 3.9–8.8% in Afro-Caribbean people from Barbados and St. Lucia [7, 8]. Recent advancements in knowledge and technology have identified that earlier cataract surgery, lens extraction, and microinvasive glaucoma surgery can prevent blindness from glaucoma [9]. These approaches are also safe in glaucoma patients with clear lenses [10].

## Primary Open-Angle Glaucoma (POAG) and Angle-Closure Glaucoma

The most identifiable cause of primary open-angle glaucoma is the age-related enlargement of the lens. The thickness of the lens increases with age [10]. During accommodation, there is increased pigment liberation due to posterior bowing and increased iridolenticular and zonular contact. The pigment liberated from zonular contact and rubbing has been shown to obstruct the trabecular meshwork that covers the inner wall of Schlemm’s canal, leading to increased stiffness and resistance. The increased lens size has also been shown to cause pupillary block and contribute to angle-closure glaucoma. The increased lens size also contributes to the narrowing of Schlemm’s canal [10]. Early cataract surgery can dramatically lower IOP and prevent glaucoma [10]. The rate of angle-closure glaucoma has declined in areas where cataract surgery is highly prevalent [11]. Recent advances in cataract surgery and microinvasive glaucoma surgery (MIGS) have had excellent results in POAG, lowering IOP, reducing medication burden, and stabilizing visual field [9, 10].

In most situations, loss of vision happens gradually over many years or even decades. However, in a smaller subset of individuals, vision loss can be quite rapid. When combined with the difficulties in the clinical evaluation of risk factors, diagnosis, screening, and care continue to be barriers [12]. In addition, there is a considerable percentage of individuals in whom POAG is not diagnosed until after severe and irreversible vision loss has already occurred [12]. Many patients of African descent globally do not have appropriate access to public health education or highly qualified practitioners who can effectively treat POAG and the visual loss associated with it.

## Unfulfilled Need in Public Health Education of Glaucoma

We must further educate the public about glaucoma and the importance of regular wellness visits. It is prudent to address the lack of public awareness in public health education efforts targeting glaucoma. One of the biggest problems in Africa is that most ophthalmologists practice in urban centers while about 80% of the population reside in rural areas [13]. In addition, about 90% of African patients that have glaucoma are unaware that they have the disease [14]. Glaucoma is six to eight times more common among Black Americans when compared to Whites; the disease tends to strike a decade earlier in Blacks, and Blacks are six times more likely to go blind from the disease. In addition, Black Americans are less likely to be educated about glaucoma by their ophthalmologists and, as a result, are less likely to adhere to their medication regimens than Whites [15]. We must continue to advocate for Black patients and ensure they receive the knowledge necessary to manage their glaucoma effectively. Although studies have not yet targeted glaucoma patients, studies done in the past have shown that educational interventions targeted towards African Americans positively influence the use of eyecare services [16]. Public health interventions regarding glaucoma are necessary to empower Black patients to ask more questions about the disease and its risk of development during visits to their primary care physicians. A higher level of understanding will also encourage patients to adhere to therapy and annual visits to their ophthalmologists for eye exams. Education regarding glaucoma in the African and African American population is crucial to reducing rates of glaucoma-related visual impairment and improving treatment success.

Furthermore, prevention with public health education on diet and exercise is an effective non-intraocular-related therapeutic that is not emphasized enough. Numerous diet and exercise-mediated interventions have been proven to improve neuroprotection in patients with glaucoma. According to the World Health Organization, a BMI of 30 or more is classified as obesity. An increased BMI has been shown to decrease perfusion of the choroid and ocular blood flow while increasing intraocular pressure [17]. More emphasis should be placed on a preventative health approach with healthy diets and exercises to prevent glaucoma in African Americans, as this population has the highest rates of obesity when compared to other groups in the USA and globally [18].

## Unfulfilled Needs in POAG Screening and Management

There is a need for improvement in low-cost glaucoma screening and educational methods to ensure that those whose screen results are positive follow up with an ophthalmologist. The goal of enhanced screening is to minimize false positives and negatives in addition to developing strategies to deploy screening in populations at high risks, such as Black populations, globally. Optical Coherence Tomography and Visual Field tests are great screening tools but are relatively expensive [19]. Not all medical facilities have access to the equipment needed to perform these tests. A low-cost glaucoma calculator can be adopted to enhance glaucoma screening and detect glaucoma earlier. In addition to intraocular pressure, central corneal thickness and age were considered while developing this screening tool which has higher sensitivity and specificity [20]. Since nonmedical individuals can perform glaucoma screening with less expensive tonometers and pachymetry, this potentially provides an affordable method for widespread glaucoma screening.

Electronic medical records (EMR) and telemedicine for glaucoma management should also be improved to be viable for clinical practices so that patients can easily follow up with an ophthalmologist after a positive screening result. EMRs should make data and tests easily available to patients and be accessible as they may travel to different physicians for eye or other medical care. In addition, investment in telemedicine is a strategy that should be promoted to reach patients in rural areas of Africa and the Caribbean who have decreased access to ophthalmologists. However, this should not replace the training of more Black and Brown ophthalmologists to meet the needs of Black patients globally.

It is essential that robust genetic testing for POAG susceptibility, inception, and advancement is developed. Genetic testing for early disease detection and identifying patients at the greatest risk of progression is ideal. When applied appropriately, genetic testing will allow physicians to identify patients who might carry genes that cause glaucoma so that they can be treated earlier to minimize sight loss [21]. Additionally, with genetic testing, ophthalmologists can tailor surveillance and testing needs for patients who carry the same genes as their relatives diagnosed with glaucoma. We should also increase diversity in genetic testing. Currently, 96% of all genetic data comes from people of European descent, even though they only make up about 15% of the global population [22]. In addition, White people make up the vast majority (79.3%) of geneticists. In comparison, just 4.1% of the population is of Hispanic or Latino origin, while 12.4% of the population is of Asian ethnicity [23]. We should strive to train more Black geneticists.

## Unfulfilled Needs in Understanding the Pathophysiology of Glaucoma

Although lowering IOP is the only accepted treatment for the disease, there are many patients with glaucoma who never present with an increased IOP [24]. There are other factors that can also play a role in glaucoma worsening despite lower IOP. This subset of factors contributes to normal tension glaucoma. Normal tension glaucoma is an optic neuropathy that can present with maximum intraocular pressure below 21 mmHg, retinal nerve fiber layer thinning, and open anterior chamber angles on gonioscopy [25]. The main causes of normal tension glaucoma are over-treated systemic hypertension, migraines, nocturnal systemic hypertension, Raynaud’s phenomenon, enlarging lens, and low vascular perfusion [25]. For example, Raynaud’s phenomenon presents with cold distal extremities as a response to cold or stress which is indicative of impaired vasoregulation. Compromised vasoregulation in Raynaud’s phenomenon and migraines have a stronger association with normal tension glaucoma than primary open-angle glaucoma [26].

Another example is Alzheimer’s disease. As many as 30% of Alzheimer’s patients have glaucoma with loss of retinal ganglion cells; many of them have normal eye pressure. Studies have shown that cataract surgery can improve and delay Alzheimer’s. A study found that participants who underwent cataract removal had almost a 30% reduction in risk of developing dementia when compared with participants without cataract removal [27].

Since other factors can play a significant role in POAG susceptibility, onset, and progression, a thorough understanding of factors besides intraocular pressure needs to be established. Further research into these factors includes but are not limited to vascular perfusion, biomolecular pathways, cerebrospinal fluid pressure, genetic history, ocular biomechanics, and retinal ganglion cell soma/axonal health. Finally, safe and accurate technologies that can be used to measure intraocular pressure over a 24-h span and assess visual and retinal ganglion cell function would be ideal for high-risk patients [21].

## Unfulfilled Needs in POAG Therapeutics

Therapeutics for lowering IOP have traditionally been topical medications (AAO preferred practice pattern) and, more recently, Selective Laser Trabeculoplasty [28]. Laroche et al. demonstrated superior treatment with clear lens extraction and intraocular lens placement in Black patients [29]. With this early surgical approach, 80% of patients no longer needed medications after 1 year with stabilization of visual field. Some patients had improvement in visual field. This approach to POAG has shown significant improvement; thus, earlier cataract surgery and MIGS should be considered as an initial approach to glaucoma by experienced surgeons. Eye drop medications such as prostaglandin analogs, alpha-adrenergic agonists, and beta blockers have traditionally been referred to as a first-line treatment for patients diagnosed with glaucoma. While these medications have been proven to reduce the rate of visual field loss, unfortunately, they do not fully halt the progression of glaucoma. Many patients find adherence to multiple glaucoma eyedrops difficult, and with increased missed doses, there is an increased progression of visual field loss [30]. There is also a global wealth gap between Blacks and Whites. These financial inequalities, in addition to other factors, make it extremely difficult for Blacks to adhere to the medication regimens prescribed by their physicians; this is a global issue. The adoption of early cataract surgery and MIGS can reduce the burden these medications have on patients, improving their quality of life while slowing visual field loss. Minimally invasive glaucoma surgery is also safer surgery to lower IOP than trabeculectomy. It offers faster visual recovery and smaller numbers of secondary interventions, such as suture lysis, and less complication. Recent trials have suggested that lens extraction through cataract surgery is even more effective in lowering intraocular pressure compared to peripheral iridotomy [31].

In resource-poor areas, microinvasive glaucoma surgery can be performed with a 23-gauge cystotome or Sinskey Hook [20]. The less expensive (MSICS) manual small incision cataract surgery is also common in such areas. The intraocular pressure (IOP) in 123 out of 147 eyes (or 83.67%) of Black patients who had manual small incision cataract surgery decreased after the procedure. In patients with glaucoma, the mean intraocular pressure (IOP) before surgery was 23.16 ± 5.68 mmHg, and the mean intraocular pressure (IOP) after surgery was 14.5 ± 2.7 mmHg, representing a 37.39% drop. This procedure is more affordable in resource-poor areas than phacoemulsification and is very effective in lowering IOP in patients with glaucoma. Instead of medication, early cataract and microinvasive glaucoma surgery should be further researched as a potential, new standard first-line treatment for patients with age-related enlarged lens–induced. As surgeons’ skills continue to improve, cataract surgery has become much safer than in the past and should be considered much earlier in patients with glaucoma to control vision loss.

For patients who might not be ideal candidates for surgery or need additional treatment after surgery, we should develop methods to improve patient compliance and medication adherence, with a focus on sustained-release drug delivery systems that reduce the need for patient compliance/adherence. Other non-IOP-related therapeutics besides patient education and diet should be developed with a focus on neuroprotection, ocular blood pressure, ocular perfusion maintenance, neural regeneration, and biomechanical reinforcement.

## Unfulfilled Needs in Patient Outcomes and Quality of Life

Preservation and maintenance of the quality of life of patients with POAG is the goal of clinical glaucoma management. From this perspective, it is prudent that vision rehabilitation among patients with glaucoma-related vision loss is improved. This will directly impact the quality of vision in patients with clinically relevant POAG progression. A standardized glaucoma-specific quality-of-life questionnaire should also be developed to aid in evaluating the true impact of this disease on patients. This quality-of-life questionnaire can also help to standardize current patient needs and further research efforts.

## Unfulfilled Needs in Physician Representation

To meet the unmet needs of Blacks with glaucoma globally and enhance the performance of early cataract surgery and MIGS, we also need to increase the number of ophthalmologists. In addition, we should train more ophthalmologists from Black and other indigenous backgrounds that are underrepresented. The 49 countries of sub-Saharan Africa are home to 12% of the global population and 23% of the global disease burden [32]. Out of 200,000 ophthalmologists in the world today, there are only about 2700 in sub-Saharan Africa, a ratio of 2.7 ophthalmologists per million population [33]. Lack of representation is also a problem African Americans in the USA are burdened by. Ophthalmology has one of the lowest representations of Black/African Americans among all medical specialties. The US population of Black individuals is about 13%; however, just 2.5% of ophthalmologists in the USA are Black [34]. We should improve the development of clinician-scientists while training more Black and other physicians of color in the USA and globally. The lack of Black and Brown physicians contributes to disparities in healthcare that afflict minorities. Studies have shown that Black patients are more likely to agree to invasive screening tests and have better health outcomes when cared for by a physician of color [35, 36].

The public should be further educated about glaucoma and the importance of scheduling annual clinical wellness visits to be screened for the disease. Regarding the field of ophthalmology, we urge researchers, clinicians, and educators to apply strategies that can increase the number of excellent surgeons to provide earlier affordable sight-saving cataract and microinvasive glaucoma surgery in most patients with age-related enlarged lens–induced glaucoma.

## Unfulfilled Needs in Patient Education

The Glaucoma Research Foundation, the National Eye Institute, and the Office of Women’s Health at the National Institutes of Health have all highlighted the need for improving the understanding of glaucoma in African American patients to reduce the disproportionate outcomes of the disease [37].

A recent study found that 67% of Black patients with glaucoma preferred that an educational program about glaucoma be offered at their physician’s office, while 39% of patients preferred it to be offered at a community or senior citizen center [37]. This simple exercise can make a big difference in communities disproportionally affected by glaucoma. Additionally, patients under the age of 70 were significantly more likely to prefer a program on the internet [37]. Internet-based education can make this information easily accessible and increase access to information that can be easily understood by patients from all backgrounds. In addition to internet-based education, increased coverage is needed in the local press, local radio, television commercials, and social media [38]. However, while the development of internet education is ideal, we must also realize that barriers still exist for those who do not have internet access.

One of the biggest reasons glaucoma disproportionately affects Blacks is the lack of knowledge this population has about the disease. Because of this, we need to invest in increased screening and educational efforts targeting this population through Black-owned media.

## Global Financial and Health Inequality

Global financial health inequality and inequity also provide many challenges to delivering eyecare services to prevent blindness from glaucoma. There are wealth gaps in the USA. According to the US federal reserve, the median wealth for a White family compared to a Black family is $188,200 and $24,100, respectively [39]. Poverty rates are still high in the Caribbean population, which has a higher prevalence of glaucoma [40]. In Africa, which also has high cases of blindness from glaucoma, the average net wealth per family is $1900 compared to $27,000 worldwide [41].

What has caused this global inequity? Reasons for this go back to the seventh century with the Arab Slave Trade [42, 43], the European Doctrine of discovery [42–44], and the papal bull “Dum Diversas” by Pope Nicholas V [45]. Further research into this history and subsequent consequences are required.

## Unfulfilled Needs for Reparations

In the movement to improve access to eyecare and prevent blindness from glaucoma, we also have to dismantle the legacy of wealth and health inequalities Reconciliation, the goal of restorative justice, requires acknowledging and dealing with the harm that was committed. This recognition then offers the beneficiaries the opportunity to restore the harm done as much as possible. Reparation was provided by the German government after World War II [46, 47]. The US government provided compensation to Japanese Americans for wrongful incarceration during World War II [48].

A 10-point reparations proposal was approved by the National African American Reparations Commission (NAARC), which is made up of Black academics and activists, during the 2015 Congressional Black Caucus annual meeting. A public expression of regret and an established Maafa/African Holocaust Institute were a part of this plan. In addition, the right to property for economic and social advancement, financial support for cooperative businesses, the creation of socially conscious entrepreneurial endeavors and resources for the healthcare, welfare, and rehabilitation of Black communities and families were all included [49]. The field of ophthalmology can also help to address these unmet needs. A plan should include, among other things, the following:

- Recruiting more Black students into medicine, ophthalmology, the ophthalmic medical industry, optometry, and the optical industry
- Providing paid scholarships for Black high school and college students to expose them to ophthalmology and optometry
- Providing ophthalmology, optometry, and ophthalmic industry research scholarships to Black medical students and residents in ophthalmology
- Recruiting, partnering with, and supporting Black ophthalmologists in leadership positions in ophthalmology, optometry, and the ophthalmic industry
- Eliminate standardized testing barriers and discrimination based on racial and wealth disparities starting at the elementary school level

## Conclusion

As research and technology in the field progress, it is prudent that public health policy and coverage standards develop in parallel. We already have many new and safer surgical techniques for the prevention and early treatment of glaucoma. We should invest in education, training, and research and address global wealth and health inequalities to expand the best ways to make the above treatments more affordable and accessible worldwide to prevent blindness from glaucoma. Particular attention needs to be placed on higher-risk persons of Black African descent globally.
